# Use machine learning models to identify and assess risk factors for coronary artery disease

**DOI:** 10.1371/journal.pone.0307952

**Published:** 2024-09-06

**Authors:** Mingyang Zhang, Hongnian Wang, Ju Zhao

**Affiliations:** 1 School of Management, Jinan University, Guangzhou, China; 2 The Second Affiliated Hospital of Xinxiang Medical University, Xinxiang, China; 3 Henan Mental Hospital, Xinxiang, China; Chinese Academy of Sciences, CHINA

## Abstract

Accurate prediction of coronary artery disease (CAD) is crucial for enabling early clinical diagnosis and tailoring personalized treatment options. This study attempts to construct a machine learning (ML) model for predicting CAD risk and further elucidate the complex nonlinear interactions between the disease and its risk factors. Employing the Z-Alizadeh Sani dataset, which includes records of 303 patients, univariate analysis and the Boruta algorithm were applied for feature selection, and nine different ML techniques were subsequently deployed to produce predictive models. To elucidate the intricate pathogenesis of CAD, this study harnessed the analytical capabilities of Shapley values, alongside the use of generalized additive models for curve fitting, to probe into the nonlinear interactions between the disease and its associated risk factors. Furthermore, we implemented a piecewise linear regression model to precisely pinpoint inflection points within these complex nonlinear dynamics. The findings of this investigation reveal that logistic regression (LR) stands out as the preeminent predictive model, demonstrating remarkable efficacy, it achieved an Area Under the Receiver Operating Characteristic curve (AUROC) of 0.981 (95% CI: 0.952–1), and an Area Under the Precision-Recall Curve (AUPRC) of 0.993. The utilization of the 14 most pivotal features in constructing a dynamic nomogram. Analysis of the Shapley smoothing curves uncovered distinctive “S”-shaped and “C”-shaped relationships linking age and triglycerides to CAD, respectively. In summary, machine learning models could provide valuable insights for the early diagnosis of CAD. The SHAP method may provide a personalized risk assessment of the relationship between CAD and its risk factors.

## Introduction

Coronary artery disease (CAD), a prevalent form of heart disease (HD), arises when the coronary arteries (i.e., blood vessels), which supply oxygen and nutrients to the heart muscle, become narrowed or blocked [1]. According to the Global Health Estimates 2019 by the World Health Organization (WHO), HD has been the primary cause of death for the past 20 years, remaining responsible for approximately 9 million deaths in 2019, corresponding to 16% of all deaths [2]. The pathogenesis of CAD is complex, primarily driven by atherosclerosis within the coronary arteries [3]. This condition is characterized by the accumulation of cholesterol and calcium deposits on the vessel walls, leading to the formation of atherosclerotic plaques that impede blood flow to the heart [3, 4]. Other predisposing factors include age, gender, hypercholesterolemia, smoking, hypertension, and diabetes [5, 6]. Although CAD is considered to have a multifactorial etiology, there is limited knowledge regarding how different factors contribute to risk. CAD brings a series of serious psychological and economic burdens for affected individuals. It is necessary to develop predictive models to identify risk factors for CAD and to evaluate the relationship between potential risk factors and CAD. This would provide diagnostic aids for physicians in clinical practice and further save patients’ lives.

Machine learning (ML) models, grounded in data-driven methodologies, have demonstrated significant success within the medical field, offering novel insights into clinical diagnosis [7]. These ML approaches present a distinctive analytical framework for the development of cost-effective interventions and advancements in disease prevention [1, 8], Furthermore, they hold the potential to identify key factors and conduct risk assessments [9]. Despite the absence of a benchmark for comparing and analyzing machine learning features, methods, and algorithms in CAD diagnosis [10], multiple studies have corroborated the advantages of models based on machine learning approaches, such as [11–16]. Alizadehsani et al. [17] applied a feature engineering method to increase model performance, compared with other methods, SVM achieved the highest AUC (0.92). Cüvitoğlu [18] used Principal Component Analysis to reduce the dimensions of the feature space and built an ensemble learner, which achieved an AUC of 0.83. Zhang et al. [19] applied five different class balancing techniques to balance the dataset, with the highest AUC (0.93) obtained by LightGBM using all features. Sayadi et al. [20] compared the performance of six ML models using three feature selection methods, and the results showed that both SVM and LR had nearly the same AUC at 0.98. Suryani et al. [21] proposed a feature selection method considering costs, with the DNN (Deep Neural Network) model achieving an AUC of 0.973 with 20 features. Das et al. [22] adopted the minimum Redundancy Maximum Relevance (mRMR) feature selection method, and the random forest (RF) model achieved an AUC of 0.911 based on 21 features. These models still have the potential to further enhance performance. Other literatures, such as [11, 17, 23, 24], focuses on improving accuracy but doesn’t report AUROC or AUPRC, which may be misleading and less credible in imbalanced datasets. While many machine learning models (e.g., random forest (RF) and XGBoost, etc.) excel at predictive performance, their decision-making processes are difficult to interpret [25], which may hinder their application in practical clinical settings. A popular and transparent model interpretability method is SHAP (SHapley Additive exPlanations), which is widely used in the existing literature [26–29]. Understanding the potential reasons for a prediction model can guide and help clinicians understand the basis of decisions. Moreover, critical risk variables that affect the development of CAD have not received enough attention.

Therefore, careful investigations of CAD risk factors and comprehensive risk assessment are necessary. This study aimed to identify critical risk factors and establish a predictive model for CAD. Recognizing that different ML algorithms may exhibit varied efficacy across specific problems, we employed a diverse array of ML techniques to construct a risk prediction model. The performance of these models was meticulously compared to determine the ML model offering optimal accuracy and clinical utility. Additionally, we utilized the SHAP approach to elucidate the nonlinear relationships between risk factors and CAD and to assess the inflection points within these relationships. To our knowledge, few studies have explored this topic using Shapley smoothing curves fitting.

## Methods

### Data resources

The Z-Alizadeh Sani dataset, designed for CAD diagnosis, encompasses records from 303 patients collected at the cardiovascular center of Shahid Rajaei Hospital. This dataset, comprising 59 features and is categorized into 216 CAD instances, with the remainder classified as healthy. These features are segmented into four categories: demographic, symptom and examination, ECG (electrocardiogram), laboratory test and echo (echocardiogram). Each patient’s diagnosis falls into one of two potential categories: Normal or CAD. Diagnostic angiography of three arteries—the left anterior descending (LAD), left circumflex (LCX), and right coronary artery (RCA)—was conducted. A diagnosis of CAD was confirmed if at least one of these arteries exhibited stenosis, defined as narrowing of at least 50%. Patients not meeting this criterion were classified as Normal. This dataset is freely accessible through the UCI Machine Learning Repository for academic research purposes.

### Data preprocessing

The dataset contained no missing values. To address potential outliers, the Interquartile Range (IQR) method was employed. The IQR is defined as the difference between the third quartile (Q3) and the first quartile (Q1). Data points falling below Q1-1.5IQR or above Q3+1.5IQR were considered outliers and replaced with Q1-1.5IQR or Q3+1.5IQR, respectively. Prediction targets were CAD onset (Yes/No), LAD, LCX, and RCA were used to determine CAD, so we removed these attributes. Feature selection was performed to extract representative feature subsets from the original data, aiming to build models with optimal performance while reducing computational costs. Due to potential correlation between length, weight, and BMI, length was deleted. The constant feature "Exertional.CP" (Exertional Chest Pain) was also removed as it provided no discernible information. Univariate feature analysis (i.e., T-test and Chi-square test) and Boruta [30] feature importance were used for selecting relevant features. Boruta is a feature selection algorithm that performs feature selection by assessing the significance of original features against randomly generated shadow features. Feature importance is determined based on the Z-score of its importance measure compared to the importance of shadow features. More important attributes receive higher importance scores. Boruta leverages the random forest classifier’s principle of adding randomness to the system and aggregating results from multiple random samples. This approach mitigates the misleading effects of random fluctuations and identifies truly relevant features.

While SHAP analysis offers significant insights into feature importance and impact, it does not directly quantify the relationships between variables. Recognizing this limitation, we incorporated generalized additive models (GAMs) to fit curves between variables and their corresponding SHAP values. This methodological enhancement allows for a more nuanced exploration of how features influence model predictions. The application of GAMs in our analysis serves two primary purposes. Firstly, it enables the identification of critical points where the SHAP value (y) equals zero for a given feature value (X). This critical point is significant because it marks a threshold beyond which the feature’s contribution to the model’s outcome may change direction—from positive to negative or vice versa. Secondly, GAMs help identify inflection points on the curve, signaling a change in the relationship between a feature and the model’s outcome. Specifically, these inflection points indicate where the odds ratio (OR), relative risk (RR), or hazard ratio (HR) values before and after the point differ. Incorporating GAMs for curve fitting thus allows us to uncover and interpret key points in the relationship between features and their SHAP values, offering a more detailed and nuanced understanding of feature contributions. This approach enhances the interpretability of machine learning models, particularly in the context of our study, by providing a clearer picture of how individual features influence model predictions.

### Model development and evaluation

The dataset was randomly partitioned into training and testing sets at an 8:2 ratio for multi-model analysis on the selected features. To mitigate the influence of randomness on outcomes, 10-fold cross-validation was employed. The average performance on the test set was calculated. Recognizing the specificity of ML algorithms to particular problem domains, we explored nine ML models to identify the optimal predictive framework: Logistic Regression (LR), eXtreme Gradient Boosting (XGBoost), Adaptive Boosting (AdaBoost), Random Forest (RF), C5.0, Support Vector Machine (SVM), K-Nearest Neighbor (KNN), Artificial Neural Network (ANN), and Naïve Bayes (NB). To address class imbalance, the Youden Index (sensitivity + specificity—1) was used to determine the optimal prediction probability cutoff. Model discrimination was evaluated using the Area Under the Receiver Operating Characteristic Curve (AUROC) and the Area Under the Precision-Recall Curve (AUPRC), facilitating a nuanced assessment of model performance in imbalance datasets. Additionally, decision curve analysis (DCA) and model calibration were conducted. SHAP analysis quantified each risk factor’s contribution to predictions, while the Partial Dependence Plot (PDP) illustrated the relationship between the target variable and features of interest. A generalized additive model (GAM) elucidated nonlinear risk factor correlations with the target, enhancing understanding of threshold and saturation effects. Piecewise linear regression was used to identify inflection points in nonlinear trends, segmenting continuous risk factors into subgroups based on these points.

### Statistical analysis

All descriptive statistical analyses were performed using R version 4.2.0. Patients were divided into two groups based on whether they had CAD. Continuous variables were displayed as means and standard deviations (SD) using the T-test, categorical variables were expressed as frequencies and percentages using the chi-square test, *p* < 0.05 was considered statistically significant. Boruta was used to capture and rank the importance of features with respect to the outcome variable. Univariate and multivariate logistic regression were used to build crude and adjusted models, respectively, along with further trend analysis.

## Results

### Characteristics of baseline data and the selection of critical factors

**Fig 1:**
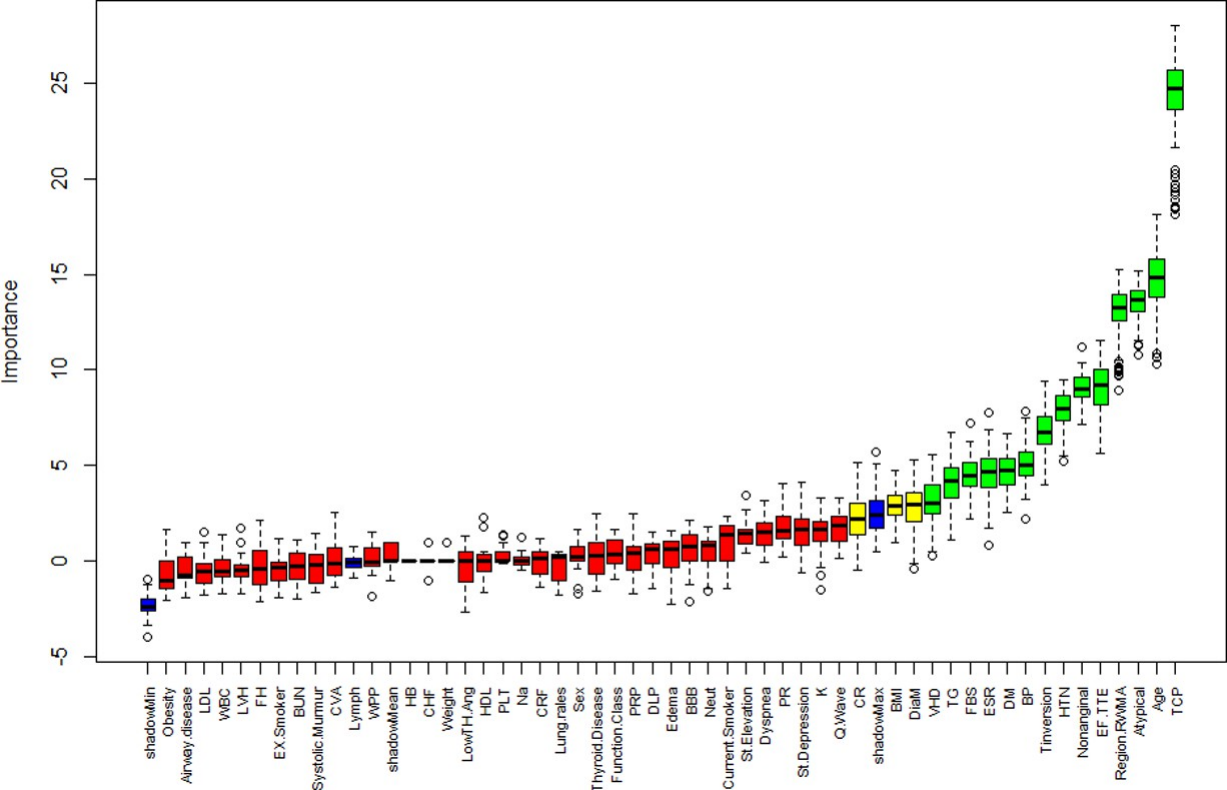
Feature selection using Boruta algorithm: Variable importance plot. Red, yellow, green, and blue boxplots represent Z scores of rejected, tentative, confirmed and shadow attributes respectively. Shadow (minimum, mean, and maximum) features are reference points for deciding which attributes are truly important and these values are generated by the algorithm via shuffling values of the original attributes. Variables extracted from the 14 confirmed important, 36 confirmed unimportant and 3 tentative features. Weak Peripheral Pulse: WPP; Diastolic Murmur: DiaM; Typical Chest Pain: TCP; Poor R Progression: PRP.

**Table 1** presents the distribution of characteristics for the study cohort, which was comprised of 216 CAD and 87 Normal. The average age of the CAD population was older than that of the normal population (53.24±8.93), there was 130 (60.2%) males, which was higher than that of females. The Boruta algorithm was used to rank features importance, as shown in **Fig 1**. The presence of multiple features with negative importance values indicates that these features were deemed less significant than the average shadow feature and were consequently excluded from the final model by the Boruta algorithm. 14 variables (green boxplots) were confirmed to be important according to the Boruta feature selection method. Therefore, a total of 14 variables were selected for modeling, considering both the p-values from univariate analysis (*p* < 0.05 in **Table 1**) and the results of the Boruta. The final feature set comprised 6 numeric variables: Age, BP, FBS, TG, ESR, EF.TTF, and 8 categorical variables: DM, HTN, Typical Chest Pain, Atypical, Nonanginal, T inversion, Region RWMA, VHD.

**Table 1:** Distribution of baseline characteristics of the dataset.

Variable	CAD (N = 216)	Normal (N = 87)	p.value
Numeric variables (mean (SD))			
Age	61.22 (9.76)	53.24 (8.93)	<0.001
Weight	73.26 (11.59)	75.05 (12.09)	0.233
BMI (Body Mass Index)	27.02 (3.86)	27.76 (4.41)	0.148
BP (Blood Pressure)	132.32 (18.21)	122.48 (18.29)	<0.001
PR (Pulse Rate)	76.00 (8.50)	72.90 (7.82)	0.004
FBS (Fasting Blood Sugar)	125.18 (52.77)	102.41 (34.72)	<0.001
CR (creatine)	1.07 (0.28)	1.02 (0.19)	0.13
TG (Triglyceride)	155.13 (81.80)	128.88 (75.31)	0.01
LDL (Low density lipoprotein)	104.08 (35.07)	105.83 (33.84)	0.692
HDL (High density lipoprotein)	39.78 (9.00)	40.65 (10.33)	0.465
BUN ((Blood Urea Nitrogen)	17.78 (6.78)	16.55 (6.11)	0.143
ESR (Erythrocyte Sedimentation rate)	21.16 (16.04)	15.01 (13.70)	0.002
HB (Hemoglobin)	13.11 (1.64)	13.26 (1.50)	0.466
K (Potassium)	4.28 (0.45)	4.10 (0.38)	0.002
Na (Sodium)	140.79 (3.80)	141.51 (3.35)	0.125
WBC (White Blood Cell)	7660.02 (2462.95)	7275.72 (2011.57)	0.197
Lymph (Lymphocyte)	31.61 (9.96)	34.38 (9.44)	0.027
Neut (Neutrophil)	60.97 (10.11)	58.17 (9.48)	0.027
PLT (Platelet)	218.22 (51.09)	226.40 (54.58)	0.217
EF (Ejection Fraction).TTE	45.93 (8.87)	50.57 (7.79)	<0.001
**Categorical variables (%)**			
Sex = Male	130 (60.2)	46 (52.9)	0.299
DM (Diabetes mellitus) = Y	80 (37.0)	10 (11.5)	<0.001
HTN (Hyper tension) = Y	147 (68.1)	32 (36.8)	<0.001
Current.Smoker = Y	49 (22.7)	14 (16.1)	0.261
EX.Smoker = Y	8 (3.7)	2 (2.3)	0.792
FH (Family History) = Y	36 (16.7)	12 (13.8)	0.656
Obesity = Y	149 (69.0)	62 (71.3)	0.8
CRF (Chronic Renal Failure) = Y	6 (2.8)	0 (0.0)	0.265
CVA (Cerebrovascular Accident) = Y	4 (1.9)	1 (1.1)	1
Airway.disease = Y	10 (4.6)	1 (1.1)	0.26
Thyroid.Disease = Y	4 (1.9)	3 (3.4)	0.679
CHF (Congestive Heart Failure) = Y	1 (0.5)	0 (0.0)	1
DLP (Dyslipidemia) = Y	79 (36.6)	33 (37.9)	0.928
Edema = Y	10 (4.6)	2 (2.3)	0.538
Weak.Peripheral.Pulse = Y	5 (2.3)	0 (0.0)	0.351
Lung.rales = Y	9 (4.2)	2 (2.3)	0.655
Systolic.Murmur = Y	29 (13.4)	12 (13.8)	1
Diastolic.Murmur = Y	3 (1.4)	6 (6.9)	0.029
Typical.Chest.Pain = Y	154 (71.3)	10 (11.5)	<0.001
Dyspnea = Y	87 (40.3)	47 (54.0)	0.04
Atypical = Y	40 (18.5)	53 (60.9)	<0.001
Nonanginal = Y	3 (1.4)	13 (14.9)	<0.001
LowTH.Ang = Y	2 (0.9)	0 (0.0)	0.907
Q.Wave = Y	16 (7.4)	0 (0.0)	0.02
St.Elevation = Y	14 (6.5)	0 (0.0)	0.033
St.Depression = Y	59 (27.3)	12 (13.8)	0.018
T inversion = Y	79 (36.6)	11 (12.6)	<0.001
LVH ((Left Ventricular Hypertrophy) = Y	16 (7.4)	4 (4.6)	0.525
Poor.R.Progression = Y	9 (4.2)	0 (0.0)	0.119
Function.Class			0.139
0	145 (67.1)	66 (75.9)	
1	0 (0.0)	1 (1.1)	
2	56 (25.9)	17 (19.5)	
3	15 (6.9)	3 (3.4)	
BBB (%)			0.303
LBBB	7 (3.2)	6 (6.9)	
Normal	204 (94.4)	78 (89.7)	
RBBB	5 (2.3)	3 (3.4)	
Region.RWMA (Regional Wall Motion Abnormality)			<0.001
0	134 (62.0)	83 (95.4)	
1	23 (10.6)	3 (3.4)	
2	31 (14.4)	1 (1.1)	
3	14 (6.5)	0 (0.0)	
4	14 (6.5)	0 (0.0)	
VHD (Valvular Heart Disease)			0.001
Mild	115 (53.2)	34 (39.1)	
Moderate	22 (10.2)	5 (5.7)	
Normal	76 (35.2)	40 (46.0)	
Severe	3 (1.4)	8 (9.2)	

Chi-square test and t-test were used for univariate analyses of categorical and continuous variables, respectively. *p* < 0.05 was considered statistically significant. SD, standard deviation.

### Performance comparison of different ML methods

**Fig 2:**
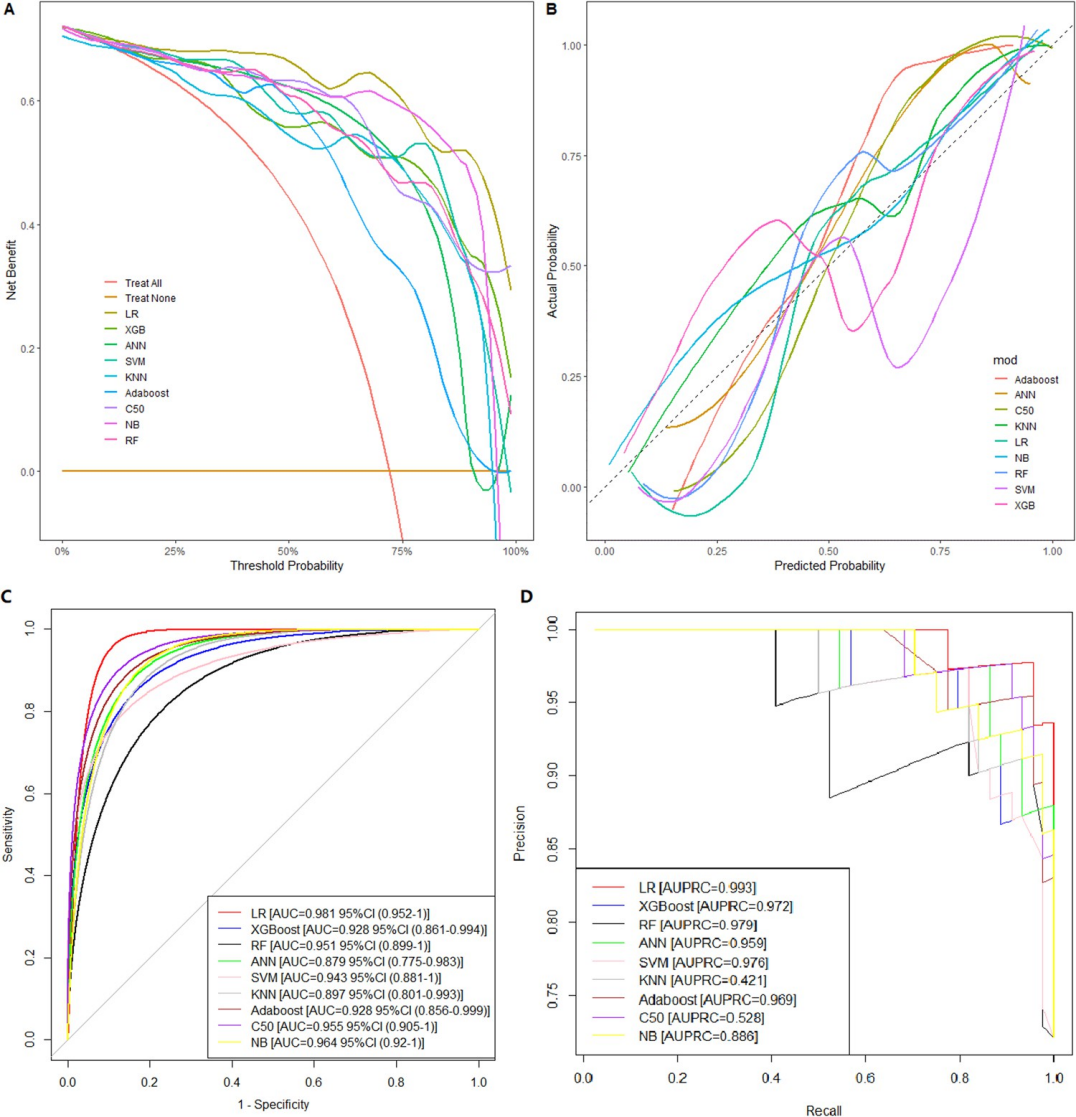
Comprehensive analysis of ML model on test set. A: Decision curves, B: Calibration curves, C: AUC (The Area Under Receiver operating characteristic Curve), and D: AUPRC (The Area Under Precision-Recall Curve).

After identifying 14 relevant factors, we divided the data into training set and test set (the ratio is 8:2). We implemented nine machine learning models (LR, RF, AdaBoost, SVM, C5.0, XGBoost, NB, KNN, and ANN) to predict CAD. Model performance was evaluated using AUPRC and AUROC, and the Decision curves and Calibration curves were also analyzed. The decision curve showed that the net benefit of all models decreases as the threshold probability increased, the LR model consistently performed best, which also proved that the LR model had the best clinical suitability (**Fig 2A**). Meanwhile, the calibration curve (The closer the Apparent line is to the dashed line, the better the agreement between the predicted and actual values is) showed that LR exhibited the best fit between the actual diagnosis and the predicted diagnosis (**Fig 2B**). And, LR also showed the highest classification capability and predictive reliability, achieving the highest AUROC (area = 0.981) and AUPRC (area = 0.993) on the test set (**Fig 2C and 2D**). Others metrics were also optimal for LR (**S1 Table)**, Accuracy, Recall and Precision were 0.951, 0.955, and 0.977 respectively. In order to prevent overfitting, a 10-fold CV was also used for training model, **Table 2** summarized the mean of model performance. LR maintained good predictive values, with an AUROC of 0.932, and showed the second highest AUPRC (0.964). RF achieved the second highest AUROC after LR, but had the highest AUPRC (0.967). Detailed evaluation metrics, including Accuracy, Recall, Precision, F1-Score and Specificity, were presented in this table.

**Table 2:** The predictive performance of nine machine learning models using 10-fold cross-validation.

Model	Specificity	Precision	F1	Recall	Accuracy	AUROC	AUPRC
**LR**	0.920	0.959	**0.925**	**0.896**	**0.901**	**0.932**	0.964
**RF**	0.958	0.979	0.912	0.857	0.888	0.931	**0.967**
**SVM**	0.947	0.975	0.917	0.869	0.891	0.919	0.963
**C50**	0.921	0.956	0.908	0.867	0.881	0.919	0.420
**AdaBoost**	0.982	0.987	0.867	0.781	0.839	0.916	0.963
**NB**	0.906	0.952	0.920	0.898	0.895	0.915	0.798
**XGBoost**	0.900	0.946	0.891	0.847	0.858	0.896	0.940
**KNN**	0.895	0.947	0.889	0.841	0.855	0.880	0.326
**ANN**	0.755	0.899	0.820	0.830	0.808	0.784	0.844

### Construction of nomgrams and risk factor assessments

**Fig 3:**
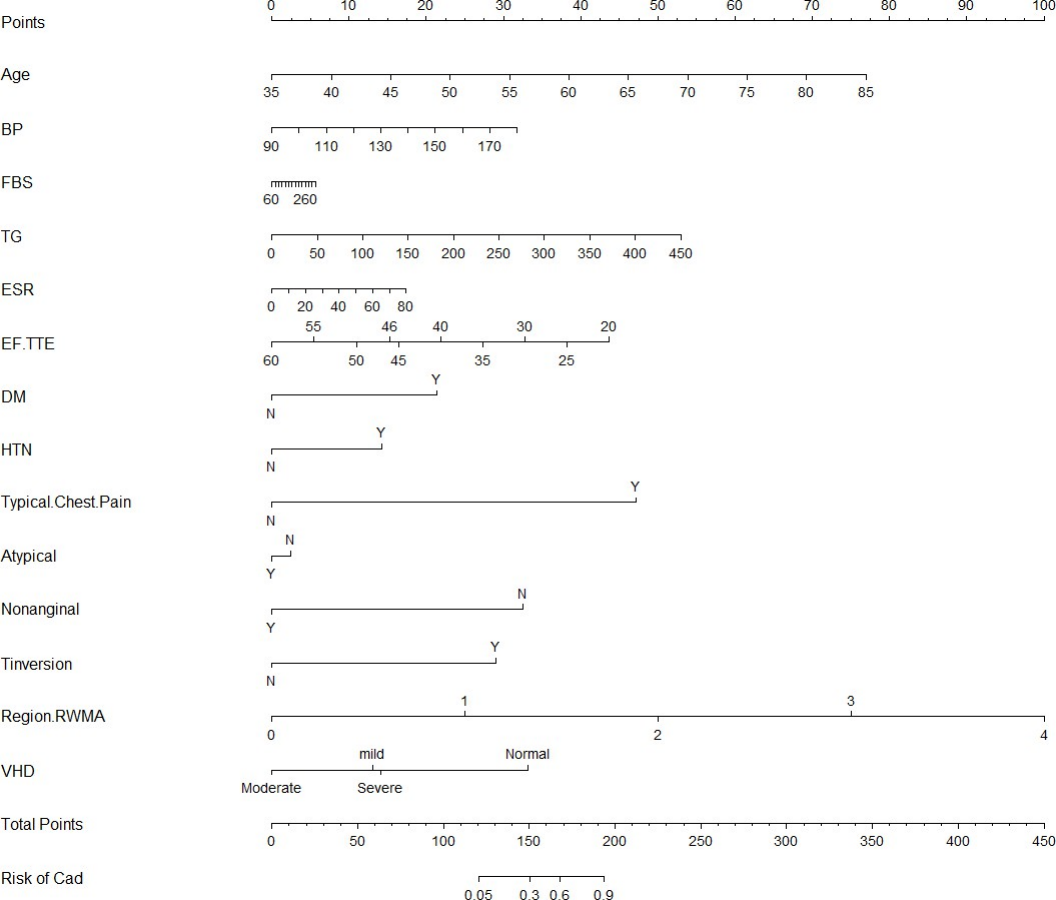
Nomogram for assessing the risk of CAD.

**Fig 4:**
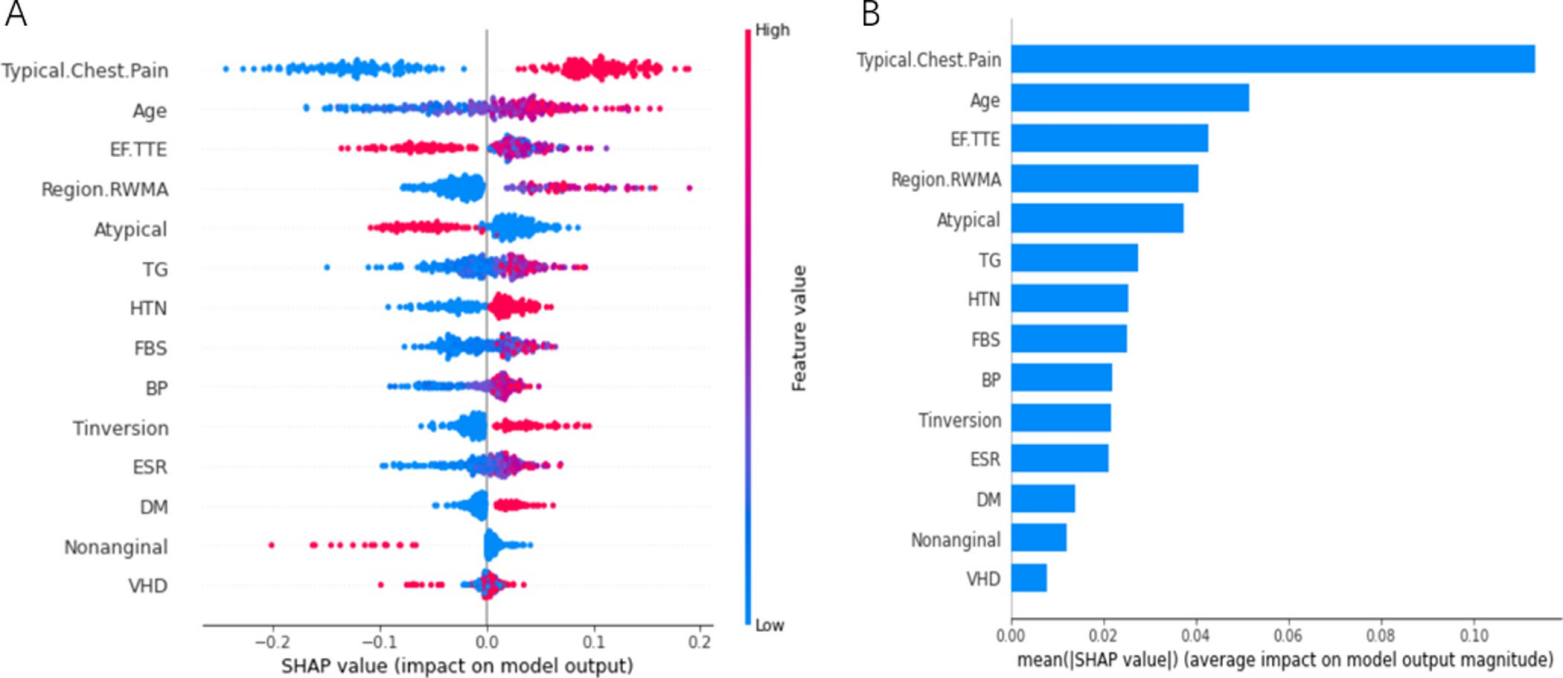
SHAP summary plot to determine the importance ranking of risk factors on the RF model.

Focused on the competitive LR model, a nomograph was constructed based on the coefficients of the variables (**Fig 3**). The risk probability of CAD can be calculated based on the sum of the patient’s scores for each factor in the nomograph. Higher scores were associated with a higher risk of CAD. The dynamic nomogram is accessible online at https://mingyangkeyan.shinyapps.io/DynNomapp/. Two models (Univariate LR and Multivariate LR) were constructed to examine the relationship between risk factors and CAD. Crude odds ratio (cOR) and adjusted odds ratio (AdjOR) were shown in **Table 3**. Age, TG, DM, Typical Chest Pain, T inversion, and Region RWMA were considered statistically significant (*p* < 0.05) in both Univariate LR and Multivariate LR model. OR values of greater than 1 for these factors suggested that the risk of CAD increases with the exposure of these factors. Among these factors, Typical Chest Pain was identified as the most dangerous factor. We noted that the cOR of VHD was less than 1, this means VHD was a protective factor, while AdjOR was greater than 1, this means that VHD may be associated with an increased risk of CAD after accounting for other potential confounders. Regrettably, the disadvantage of LR model is that the interpretation is difficult because the interpretation of the weights *β* (*OR* = *e*^*β*^) is multiplicative and not additive. Given that RF and LR have similar advantages in predictive power, and especially the discrimination (AUPRC, area = 0.967) of RF in unbalanced data by performing 10-fold. In order to provide visual explanations for the selected variables, SHAP was used to analyze RF model. **Fig 4** shows the 14 most important features. Each row represents a feature, with SHAP values for each sample plotted as different colored dots, where the red and blue dots represent high risk and low risk respectively. For example, patients with Typical Chest Pain generally have a higher risk of CAD, and the risk of CAD increases with age. Moreover, this figure also shows the feature importance ranking, the importance diminishes from top to bottom, with Typical Chest Pain exhibits the most potent predictive power and followed by Age.

**Table 3:** The analyses results of univariate and multivariate logistic regression model.

Var	Univariate LR		Multivariate LR	
	OR (95%CI)	P value	AdjOR (95%CI)	P value
**Age**	1.095(1.063–1.131)	0	1.109(1.057–1.17)	0
**BP**	1.032(1.017–1.049)	0	1.027(0.996–1.062)	0.101
**FBS**	1.013(1.006–1.022)	0.001	1(0.985–1.015)	0.979
**TG**	1.005(1.001–1.009)	0.012	1.008(1.002–1.015)	0.009
**ESR**	1.032(1.012–1.054)	0.003	1.014(0.985–1.048)	0.367
**EF.TTE**	0.925(0.888–0.959)	0	0.963(0.907–1.017)	0.192
**DM**	4.529(2.309–9.772)	0	4.89(1.232–21.945)	0.03
**HTN**	3.662(2.188–6.219)	0	2.294(0.785–6.903)	0.131
**Typical Chest Pain**	19.126(9.689–41.572)	0	32.036(8.075–146.194)	0
**Atypical**	0.146(0.083–0.251)	0	1.044(0.296–3.803)	0.947
**Nonanginal**	0.08(0.018–0.257)	0	0.137(0.016–0.937)	0.053
**Tinversion**	3.984(2.071–8.334)	0	6.562(2.271–21.465)	0.001
**Region RWMA**	4.767(2.523–12.179)	0	5.986(2.337–22.249)	0.001
**VHD**	0.672(0.522–0.86)	0.002	1.622(1.013–2.713)	0.050

**Fig 5:**
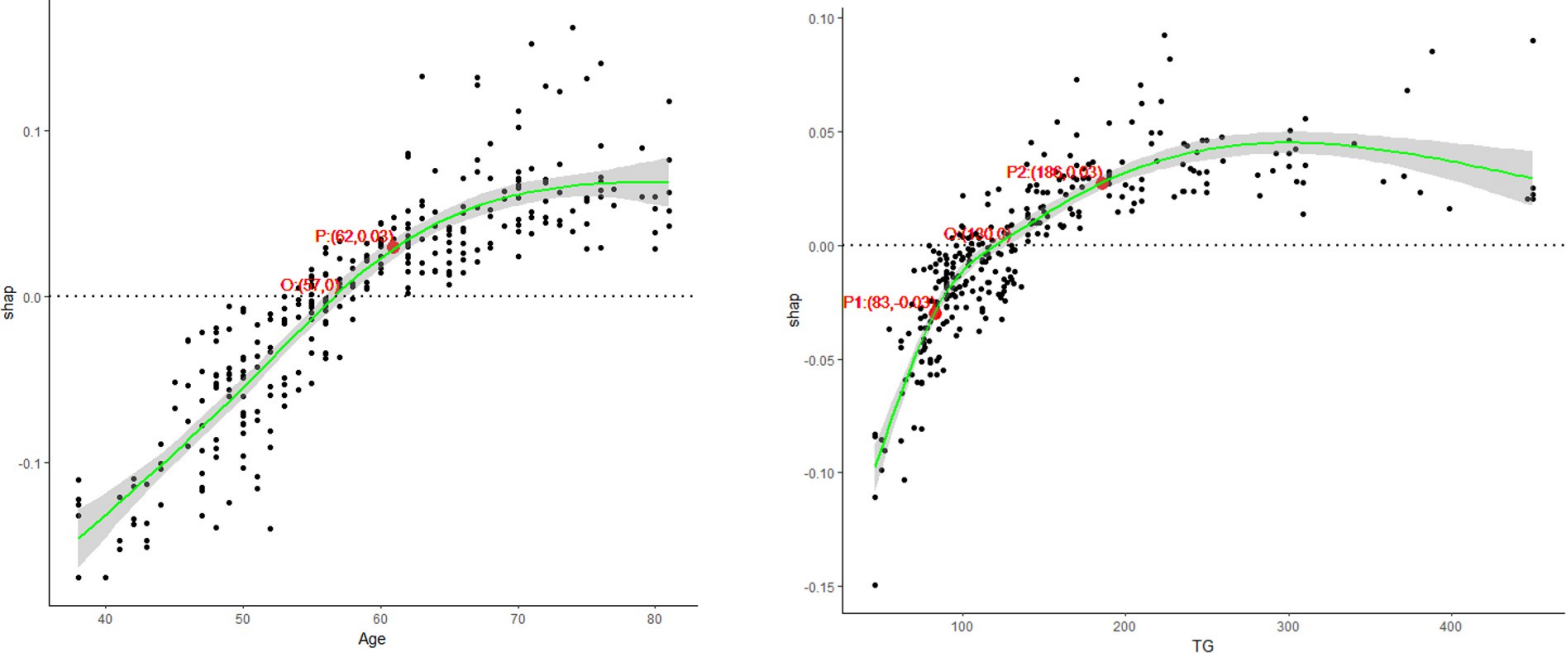
Non-linear dependent relationship between Age/TG and the risk of CAD. TG: triglyceride(mg/dl), “O” is zero point, “P”, “P1”, “P2” are the inflection point. P:(62,0.03) and O (57,0) for age, P1:(83, -0.03), P2:(186,0.03) and O (130,0) for TG.

Furthermore, we also analyzed the marginal effect on CAD risk of two continuous variables in detail using SHAP values, the PDP of Age and TG were displayed in **Fig 5**. The “S”-shaped and “C”-shaped curve were discovered on the exposure-response relationship between critical factors and CAD risk. The smooth curve fitting revealed a threshold effect and saturation effect on PDP. Initially, CAD prevalence showed an overall increasing trend with Age or TG, and then tended to flatten out. When age exceeded 57 years or TG exceeded 130mg/dl, these factors exhibited a positive effect on CAD occurrence. Further, we used piecewise linear regression to find the inflection point of the non-linear connections, and divided into subgroups according to these observe point. We found that 62 years was the inflection point for Age, and 83 and 186 mg/dl were the inflection point for TG. The effects of Age and TG at different segments on CAD risk were shown in **Table 4**. The risk of CAD significantly increased by 284% when age exceeded 62 years, with an odds ratio (OR) of 3.84 (95% CI: 2.12–7.35) compared to those aged less than 62 years. When TG ranged from 83 to 186 mg/dl, the risk of CAD increased by 60.9% compared to TG<83mg/dl, but the association was not statistically significant. When TG exceeded 186 mg/dl, the risk of CAD significantly increased from 1.609 to 3.236. Other risk factors Shapley curves are showed in **S1** and **S2 Figs.**

**Table 4:** Threshold effect analysis using piecewise linear regression model.

Variable	Groups	OR (95%CI)	P
Age	< = 62	Ref	
	>62	3.84 (2.120–7.347)	0
TG			
	< = 83	Ref	
	83∼186	1.609 (0.830–3.081)	0.153
	>186	3.236 (1.432–7.548)	0.05

## Discussion

This study identified 14 critical features for assessing CAD risk using ML models. Compared to other models in this study, LR exhibited superior discriminative capabilities. This work not only constructed a prediction models, but also investigated the nonlinear relationship between risk factors and CAD by SHAP values, including an analysis of cutoff points, an aspect that has received limited attention in previous studies. This work can not only improve the interpretability of the model but also enrich medical knowledge by quantifying the underlying mechanisms of disease development. Furthermore, this study delves into the identification of thresholds and inflection points for risk factors, empowering clinical decision-makers to devise more targeted strategies and interventions with greater precision.

Diversity is a key property of medical datasets. Although it provides rich and comprehensive information, it also significantly increases the complexity of medical data mining. To identify risk factors for CAD, extracting more informative medical knowledge is crucial for improving the accuracy and stability of predictive models. Through feature selection, we identified several crucial factors for CAD diagnosis, these features were included: 4 Demographic (Age, BP: Blood Pressure, DM: Diabetes Mellitus, HTN: Hypertension); 6 Laboratory and Echo (FBS: Fasting Blood Sugar, TG: Triglyceride, ESR: Erythrocyte Sedimentation Rate, EF TTF: ejection fraction, Region RWMA: regional wall motion abnormality, VHD: valvular heart disease); 3 Symptom and Examination (Typical Chest Pain, Atypical, Nonanginal); 1 ECG (T inversion). The current literature consistently demonstrates a relationship between these covariates and coronary artery disease (CAD). Advancements in information technology have made analyzing patients’ biochemical characteristics for CAD risk assessment increasingly feasible and cost-effective. Among the models developed using these features, Logistic Regression (LR) and Random Forest (RF) have consistently shown robust performance. Due to its simplicity and ease of application in clinical settings, we chose to develop a nomogram based on LR. This nomogram, incorporating 14 variables, aims to assist clinicians in making informed decisions regarding the management and treatment of CAD patients. Furthermore, RF achieved superior outcomes through 10-fold cross-validation, suggesting that the selected set of features effectively discriminates against CAD. This finding enhances the reliability and practical utility of our results in a clinical context.

Exploring risk factor trends is crucial for reducing the prevalence of CAD. This study focused on two continuous variables: age and triglyceride (TG) levels, aiming to elucidate how CAD risk fluctuates with changes in these parameters. We employed Shapley values to visually delineate the underlying mechanisms of CAD etiology. These insights are crucial for clinicians to evaluate the potential for adverse outcomes and to strategize preemptive interventions. The relationship between age and CAD outcomes diverges depending on the cutoff value. When age exceeded 57, a positive effect was observed, indicating a higher risk for the elderly as age increases [31, 32]. This may be associated with the deterioration of physical functions and complicated diseases. TG was considered an independent risk factor for CAD [33], high TG levels significantly increase CAD risk, with a positive effect observed when TG levels exceed 130 mg/dL. This suggests that maintaining TG levels below 130mg/dL may effectively reduce CAD risk [33, 34]. In this study, the breakpoints of the non-linear connection between TG and CAD were estimated to range from 83 to 186 mg/dL, which were inconsistent with existing literature. Indeed, TG is regulated by multi-facet factors [35, 36], these include inactivity, smoking, drinking too much alcohol, and being overweight, etc. [37]. CAD detection relies on a multitude of variables. For instance, high BP significantly elevates CAD risk [38, 39], and HTN is a major contributor to the development [39]. DM heightens susceptibility to CAD [40], and even in the absence of DM, FBS levels pose a risk. Chronic hyperglycemia can lead to arterial hardening, thereby increasing CAD risk [41]. The ESR provides diagnostic information about CAD [42], ESR was used to assess inflammatory status, which may be associated with CAD events. EF is utilized to assess cardiac function and monitor cardiovascular disease, where a reduction in EF signifies increased predictive value for CAD [43]. Additionally, Region RWMA [44], valvular heart disease [45], Typical Chest Pain [46], Atypical, Nonanginal [47], T inversion [48] have been recognized as predictors of CAD in prior studies.

This study possesses several strengths. Firstly, compared to other studies utilizing the same dataset [17–22], we achieved high performance while selecting relatively fewer features. Secondly, we introduced a dynamic nomogram for rapid and accurate assessment of CAD risk, thereby enhancing clinical decision-making capabilities. Thirdly, while prevailing machine learning investigations predominantly focus on model performance and risk factor identification, they frequently overlook the detailed influence of these factors on CAD. To address this gap, we employed Shapley values to elucidate the non-linear relationships between risk factors and CAD, shedding light on the threshold effects and contributing to a more nuanced understanding of CAD pathogenesis. Lastly, we innovatively applied a piecewise linear regression model to estimate breakpoints in these non-linear relationships, offering novel clinical insights that may inform the development of targeted intervention strategies.

This study has several limitations. Firstly, while these factors may be good predictors, clinicians are often more concerned about causation. Future research should focus on unraveling the causal relationships between these variables and CAD, which would contribute to precision medicine. Secondly, although our model offers valuable insights for clinical diagnosis, treatment, and the identification of potential risk factors for CAD, its applicability requires further validation with external datasets to confirm its generalizability and accuracy. Thirdly, the Z-Alizadeh Sani dataset is a public and single-center database. Its limited sample size may result in inadequate training of predictive models, and data class imbalances may lead to model bias. To enhance the model’s robustness and reliability, future efforts should focus on incorporating a broader and more diverse patient population and addressing the issue of class imbalances within the dataset. Finally, future research should integrate imaging data with other metadata for faster and more accurate disease diagnosis.

## Conclusion

Machine learning models are an effectively tool for predict the risk of CAD. Utilizing Shapley values and generalized additive models may reveals complex nonlinear interactions, aiding in early diagnosis and personalized treatment.

## Supporting information

S1 Table Performance for nine models in the test set. (DOCX)

S1 Fig Shapley values smoothing curves. (TIF)

S2 Fig Shapley values of the categorical variable. (TIF)
