# Supplementary material for: Use machine learning models to identify and assess risk factors for coronary artery disease
Source: PLoS One. 2024 Sep 6;19(9):e0307952. doi: 10.1371/journal.pone.0307952 (PMC11379138; doi:10.1371/journal.pone.0307952)
Supplement: S1 Table — (DOCX) [file pone.0307952.s001.docx]

**Supporting Information**

**S1 Table.** Performance for nine models in the test set.

| Model | Specificity | Precision | F1 | Recall | Accuracy | AUROC | AUPRC |
| --- | --- | --- | --- | --- | --- | --- | --- |
| LR | 0.941 | 0.977 | 0.966 | 0.955 | 0.951 | 0.981 | 0.993 |
| XGB | 0.941 | 0.972 | 0.875 | 0.795 | 0.836 | 0.928 | 0.972 |
| RF | 0.824 | 0.932 | 0.932 | 0.932 | 0.902 | 0.951 | 0.979 |
| ANN | 0.765 | 0.913 | 0.933 | 0.955 | 0.902 | 0.879 | 0.959 |
| SVM | 0.941 | 0.974 | 0.916 | 0.864 | 0.885 | 0.943 | 0.976 |
| KNN | 0.941 | 0.973 | 0.889 | 0.818 | 0.852 | 0.897 | 0.421 |
| Adaboost | 0.765 | 0.913 | 0.933 | 0.955 | 0.902 | 0.928 | 0.969 |
| C50 | 0.882 | 0.955 | 0.955 | 0.955 | 0.934 | 0.955 | 0.528 |
| NB | 0.941 | 0.976 | 0.941 | 0.909 | 0.918 | 0.964 | 0.886 |
