# Supplementary figures and images for: Use machine learning models to identify and assess risk factors for coronary artery disease
Source: PLoS One. 2024 Sep 6;19(9):e0307952. doi: 10.1371/journal.pone.0307952 (PMC11379138; doi:10.1371/journal.pone.0307952)

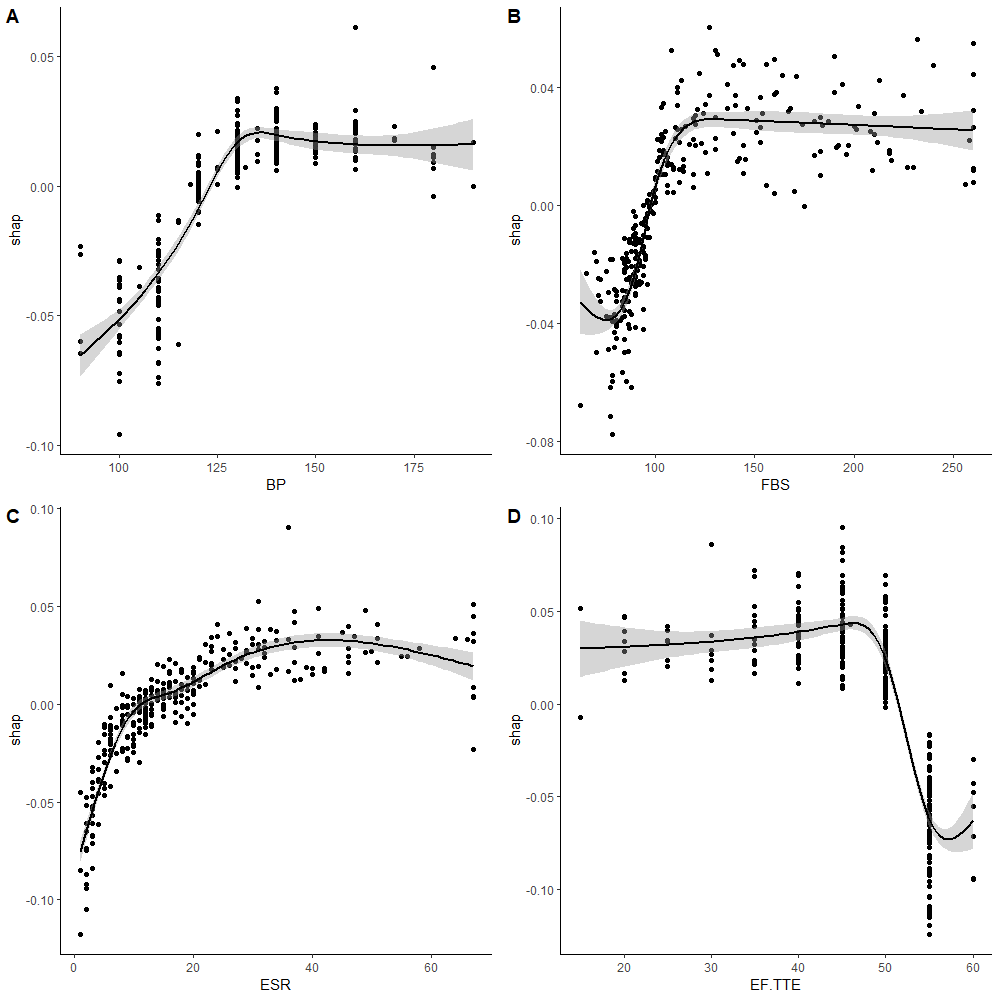

Supplement: S1 Fig — (TIF) [file pone.0307952.s002.tif]

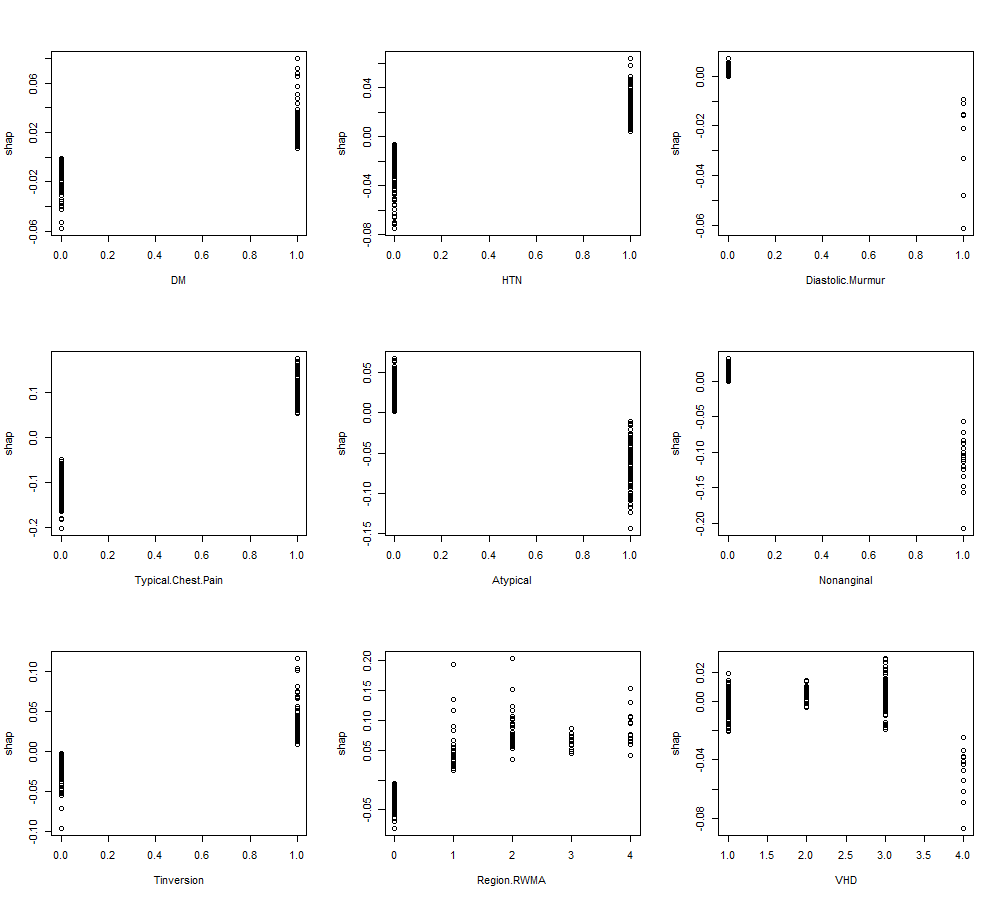

Supplement: S2 Fig — (TIF) [file pone.0307952.s003.tif]
